# Contingency Management for Patients with Cooccurring Disorders: Evaluation of a Case Study and Recommendations for Practitioners

**DOI:** 10.1155/2012/731638

**Published:** 2012-01-26

**Authors:** Claire E. Adams, Carla J. Rash, Randy S. Burke, Jefferson D. Parker

**Affiliations:** ^1^Department of Health Disparities Research-Unit 1440, P.O. Box 301402, Houston, TX 77230-1402, USA; ^2^Department of Medicine, University of Connecticut Health Center, Farmington, CT 06030-3944, USA; ^3^G.V. (Sonny) Montgomery VA Medical Center, Jackson, MS 39216, USA; ^4^Department of Psychiatry and Human Behavior, University of Mississippi Medical Center, Jackson, MS 39216, USA

## Abstract

Research indicates that contingency management (CM) has potential to improve a number of outcomes (e.g. substance use, treatment attendance, quality of life) among individuals with substance use and cooccurring disorders. However, multiple factors must be considered on a case-by-case basis in order to promote optimal treatment effects. The present study describes an individualized CM protocol for a US Veteran with substance dependence and cooccurring severe mental illness. CM targeted attendance at outpatient appointments and appropriate use of hospital resources. Effects of CM were assessed by comparing the 3-month baseline and CM periods. The CM intervention marginally reduced unnecessary hospital admissions, resulting in cost savings to the medical center of over $5,000 in three months for this individual. However, CM did not affect outpatient attendance. Several complications arose, highlighting challenges in using CM in populations with substance use and cooccurring disorders. Practical suggestions are offered for maximizing the effects of CM.

## 1. Introduction

Contingency Management (CM) is an operant-based procedure for facilitating behavior change consistent with the goals of treatment (e.g., increasing negative drug screens or decreasing violent acts [1]). CM involves rewarding explicitly defined and objectively verifiable target behaviors in an attempt to alter the frequency of those behaviors. CM can be used to elicit behavior change in situations where natural reinforcers exist for maladaptive behaviors, such as substance use. Successive attainment of goals can be rewarded through the use of bonus systems (e.g., for every three consecutive negative drug screens) in addition to the base reward system.

CM has been successfully and broadly applied in the area of substance use. While research is most prolific in the area of cocaine dependence, CM has been applied to the treatment of heroin and other opiates, cigarettes, marijuana, alcohol, and methamphetamines [2–8]. Research supports the use of CM for targeting abstinence [2, 4, 5, 9–17] and treatment attendance [10, 12, 18–22]. CM is also efficacious in promoting retention and abstinence in difficult-to-treat substance abusing populations, such as cocaine abusers with multiple past treatment attempts and cocaine abusers with comorbid alcohol dependence [23, 24]. By promoting abstinence among substance abusers, CM may improve quality of life [14].

 One of the more appealing features of CM is its utility in challenging populations, such as individuals with substance use and psychiatric disorders. For example, CM improved treatment attendance in alcohol users with cooccurring disorders [25] and opioid-dependent patients with comorbid antisocial personality disorder [26]. CM might be particularly effective for enhancing treatment attendance among individuals with higher psychiatric severity. Weinstock et al. [27] investigated relationships between psychiatric severity and treatment retention for patients who received standard treatment versus standard treatment plus CM. Increased psychiatric severity was related to lower treatment retention for individuals with substance use disorders in the standard treatment group. However, among those who received CM, this relationship was not significant, suggesting that CM may better retain those patients with greater psychiatric severity relative to standard treatments alone.

 In addition to increasing attendance, CM is efficacious in reducing substance use among patients with cooccurring disorders. In the Weinstock et al. [27] report, participants in the CM group were more likely to achieve at least 8 weeks of abstinence relative to standard treatment. This effect of CM on abstinence was similar across psychiatric severity levels. In a systematic review, Drake et al. [28] concluded that CM improves substance abuse outcomes in substance users with cooccurring severe mental illness (SMI). Messina et al. [29] reported that CM was more effective than cognitive-behavioral therapy for increasing the frequency of negative drug screens among cocaine-dependent patients with antisocial personality disorder. CM is also effective with reducing nicotine and marijuana smoking among individuals with cooccurring disorders. For example, CM reduced smoking in two within-subjects, A-B-A reversal designs studies in smokers with [30, 31]. Sigmon and Higgins [5] found that CM increased frequency of negative drugs screens among patients with SMI and marijuana dependence during a 12-week CM intervention.

 The dissemination of CM techniques into routine practice presents interesting points of consideration. Often, CM is applied to individuals who demonstrate challenging patterns of behavior and who have not met with success through other treatment avenues. That CM benefits these treatment-recalcitrant individuals points to its potential clinical utility. Petry and colleagues [32] described three clinical case studies where CM was applied in a manner individualized to the client and clinical concerns. All three cases had more than one substance use disorder and complex dual diagnoses. Target behaviors were chosen with regard to priority and case formulation (abstinence, attendance, or both). While the CM implementation had an effect on the target behavior in each of these cases, it was notable that nontarget behaviors (e.g., medication adherence, reduced psychiatric hospitalizations, improved personal hygiene) also were affected in a positive direction.

 One of the cases presented by Petry et al. [32] involved a case similar to that described in the present case study. The client was an individual with paranoid schizophrenia, multiple substance use disorders, and antisocial personality disorder. The client had a history of misusing the psychiatric emergency room for the purpose of obtaining shelter and food and often threatened suicide or was aggressive toward staff or other patients when thwarted. Due to the complexity of the case presentation, several behaviors were targeted: abstinence, attendance to scheduled appointments, not using the psychiatric emergency room other than at scheduled meetings with clinician, and medication compliance. Initially, all targeted behaviors showed remarkable change, including 17 weeks of abstinence. Although the client suffered relapses later in treatment, the authors noted that the patient continued to show improved therapy attendance, appropriate use of hospital resources, and medication compliance.

 Clearly, CM has the potential for improving target behaviors and quality of life in populations with cooccurring disorders. However, multiple factors must be considered in order to implement CM optimally. Petry [33] highlighted nine practical considerations based on learning theory to increase the effectiveness of CM. First, Petry recommended creating a very clear, understandable *behavioral contract *for each patient that outlines specific behaviors to be monitored, when and how they will be monitored, and the specific reinforcement schedule. Second, the monitored behaviors must be *objectively quantifiable and verifiable*. For example, if drug abstinence is to be reinforced, drug screening procedures must be used, rather than relying on self-report. Third, Petry emphasized the importance of *consistency* across the CM period. For example, care should be taken to avoid therapist drift in administration of the CM protocol over the intervention period. Fourth, *frequency* of the target behavior, monitoring schedule, and reinforcement opportunities should be considered; monitoring and reinforcement opportunities should occur frequently. Fifth, in some cases, *reinforcing successive approximations* of the target behavior (e.g., reduced metabolites, indicating lower drug use) may be helpful to motivate patients toward positive change in initial stages of CM. Sixth, some patients may benefit from early access to reinforcers (“*priming*”), which may solidify the relation between their participation in treatment and desirable outcomes. Seventh, reinforcers should be provided as *immediately* as possible following target behaviors (e.g., after providing a negative drug screen). Eighth, the *magnitude* of the reinforcer should be consistent with the degree of reinforcement that patients derive from the behavior to be changed. For example, too small or undesirable reinforcers are not likely to be sufficient to change drug use. Ninth, an *escalating system of reinforcers and bonuses* best promotes continued change.

 In the current study, an individualized CM program was created for a patient with cooccurring disorders. However, several barriers arose over the course of treatment, highlighting areas of improvement for future CM programs. In addition to describing the effects of CM in the present study, we sought to evaluate the individualized CM protocol in light of Petry's [33] recommendations in order to offer practical suggestions for clinicians. Specific purposes of the present study were to (1) describe a CM protocol designed for a patient with substance dependence and cooccurring SMI, (2) investigate direct effects of CM on target behaviors (weekend admissions and outpatient attendance), as well as any additional effects on related behaviors not targeted specifically by CM (e.g., results of urine drug screens), and (3) identify areas in which our CM program could have been improved in order to provide suggestions for clinicians using CM in populations with cooccurring disorders.

## 2. Case Presentation

The present case study was derived through clinical care provided in an Addictive Disorders Treatment Program at a Veterans Affairs Medical Center. This case study is based on one Veteran, a 40-year-old African American male who had repeatedly sought inpatient admissions during the weekends while in an intoxicated state. The Veteran had diagnoses of paranoid schizophrenia, alcohol dependence, and cocaine dependence.

### 2.1. Presenting Complaints

The Veteran abused alcohol and crack cocaine despite associated financial, social, and legal problems, including homelessness. He failed to attend scheduled outpatient appointments and frequently presented to the emergency department in crisis. He often sought inpatient admission to the hospital in order to avoid consequences related to his drug use (e.g., to avoid facing drug debts). These admissions were typically on weekends, likely due to a higher probability of being successfully admitted because of systemic factors (e.g., staff working on the weekend clinic who might be less familiar with his case). Thus, the Veteran generated a high number of weekend hospital admissions for detoxification, homicidal, and/or suicidal ideation. The Veteran's psychiatric difficulties and concerns regarding substance use presented significant challenges to his daily living and management of medical care.

### 2.2. History

The Veteran dropped out of school in the tenth grade and received his General Equivalency Diploma (GED) while in the army. He was the fourth of seven children raised by his mother and grandmother. He never married or had children. The Veteran was diagnosed with Schizophrenia at the age of 12 and as an adult had been hospitalized approximately 30 times for psychiatric or emotional problems. In addition, he previously attended residential substance abuse treatment for alcohol and crack cocaine on eight different occasions; however, he denied ever having a period of abstinence outside of treatment. Criminal history included at least 12 charges related to shoplifting/vandalism, burglary, larceny, and driving while intoxicated. The Veteran had a history of verbal and physical aggression towards staff. Although he was not formally diagnosed with a personality disorder, traits of antisocial personality disorder were noted in his medical chart. At the time of treatment, he was unemployed and homeless. Medical diagnoses included Herpes Simplex and history of Hepatitis A (resolved). Prescriptions included Risperidone (2 mg taken nightly at bedtime).

### 2.3. Assessment

Veteran reported drinking at least 6 beers every day and indicated that his alcohol use often led to crack cocaine use. He indicated feeling like he “cannot control [his] behavior” while drinking. He acknowledged using alcohol in greater amounts than planned, recurrent desire to control his alcohol usage, and that his alcohol use interfered with family and social functioning. Veteran reported spending $30 or more on crack cocaine per day. He acknowledged a history of using more cocaine than planned, having recurrent desire to control his usage, and that cocaine interferes with his social and occupational functioning. Specifically, his use of cocaine contributed to difficulties with self-care, relationship problems, and legal problems. He had a history of multiple drug-related arrests. Veteran also had a significant history of noncompliance with medication, including using alcohol and cocaine while being prescribed medications to treat schizophrenia.

### 2.4. Case Conceptualization

At the time of treatment, Veteran was unstable emotionally, financially, and medically. He was facing legal charges, unemployed, having financial difficulties, homeless, and not taking his medications as prescribed. Although the Veteran had entered into substance abuse treatment many times, Veteran had never attended outpatient appointments consistently or achieved sustained abstinence from substances. Instead, he sought to be hospitalized in times of crisis (typically when intoxicated and requesting detoxification). A CM program was viewed as a potentially useful tool for creating a consistent, predictable system of rewards for specific behaviors.

A CM system was developed with the goal of encouraging more appropriate use of the hospital system, and indirectly to encourage better health care decisions. The aim was not to deny (or reduce) services, but rather to encourage more appropriate use of hospital resources. As such, the target behaviors were (1) increasing engagement in scheduled outpatient appointments and (2) decreasing weekend admissions. Based on the extant literature on behavioral contracting and contingency management for implementing behavioral changes, we hypothesized that weekend hospital admissions would reduce and attendance at scheduled outpatient appointments would increase as compared to baseline.

### 2.5. Course of Treatment and Assessment of Progress

The number of admissions, type of discharge, and length of stay, as well as attendance at outpatient visits, were collected from computerized medical records. To investigate the impact of the CM procedures on abstinence from substance use, results of urine drug screening were analyzed. All data utilized in this study were collected in the process of routine clinical care. We collected retrospective data for the three months prior to implementation of the behavioral contract and for the three months while the contract was instated. A follow-up period after the behavioral contract was planned; however, this data collection was not possible due to the Veteran's incarceration, as explained later.

As part of the Veteran's individual treatment plan, a behaviorally based contract was developed for a 3-month intervention. The purpose of the contract was to facilitate communication between the Veteran and treatment team regarding the treatment plan. The contract specified two target goals and the related contingencies for each behavior. The first target goal, reducing weekend admissions, specified that he would receive coupons in the amount of $7 for not seeking admission to the hospital on weekends, defined as between 4pm Friday and 8am Monday. These coupons could be used in the Veterans' Canteen Store to buy assorted items such as snacks and appliances. The value of the coupons would increase in increments of $3 for each week of consecutive compliance up to six weeks (i.e., $7 for the first week, $10 for the second week, up to $22). Following the sixth consecutive compliant week, the amount of coupons would remain at $22. Failure to comply with the target goal resulted in no reinforcement for that week. In addition, the coupon amount was reset to the initial $7 amount for the following week and escalation resumed according to the schedule for subsequent weeks.

For the second goal of once weekly attendance at scheduled outpatient appointments, the payment schedule started at $1 for the first session and increased by $2 increments for consecutive weeks of attendance. This incremental increase was capped at $11 after the sixth week and remained at this level unless the target behavior was not met. As with the first goal, failure to meet the target goal resulted in no reinforcers for that day and the voucher amount was reset back to the initial value for the following visit.

**Figure 1:**
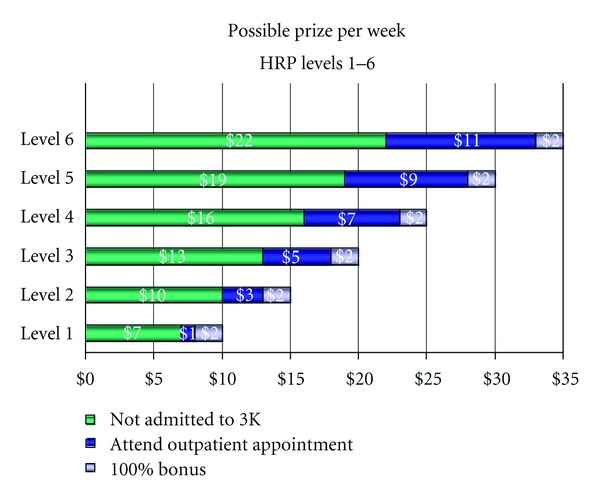
Payment schedule.

In addition to the payment schedules listed above, another bonus system was implemented to encourage completion of both behaviors. This bonus constituted an additional $2 for each month that both target behaviors were consistently met. A stipulation was added to the contract which stated that aggressive behavior would result in a warning and continued aggressive behavior would result in one of the payment schedules (either attending scheduled outpatient appointments or being admitted to the hospital over the weekend**) **being reset to the initial values. The client was provided with a table describing the payment schedule to better illustrate possible earnings (see Figure 1).

## 3. Results

**Weekend Admissions.**

Data from medical records indicated that during the 3 months before CM, the Veteran was admitted to the hospital on weekends on 4 separate occasions. During 3 months of CM, the Veteran had one-weekend hospitalization. Chi-square analysis was used to test differences in frequency of weekend hospitalization between the pre-CM and CM periods. Results indicated that frequency of weekend admissions was marginally lower during the CM period, *χ* ^2^(1) = 2.27, *P* = .07, one tailed. Cost savings associated with this reduction in weekend admissions was calculated using the average costs of care on this inpatient mental health unit ($1,121.59 per bed per day). The Veteran was hospitalized for six weekend days during the pre-CM period (totaling $6,729.54). During CM, the Veteran's cost of weekend hospitalization ($1,121.59) plus his maximum possible CM earnings ($345) summed to $1,466.59, resulting in savings of at least $5,262.95 over three months. Because administration of the CM protocol ranged between 5 and 10 minutes per week and was integrated into the case manager visits, CM did not incur significant additional costs beyond the cost of vouchers.

**Outpatient Attendance.**

During the 3 months before CM, the Veteran attended 2 of 12 scheduled appointments. During 3 months of CM, he attended 4 of 12 scheduled appointments, with no differences in percent of scheduled appointments attended between the pre-CM (16.67%) and CM (33.33%) periods, *χ* ^2^(1) = .89, *P* = .17, one tailed.

**Drug Screens.**

Veteran submitted 7 urine drug screens in the 3 months preceding CM and 3 drug screens during CM. Drug screens were scheduled randomly. Veteran's drug screens indicated that he was not abstinent from cocaine at any point before or during CM.

### 3.1. Evaluating the Individualized CM Program

Overall, the present CM program resulted in marginally fewer weekend hospitalizations and lower associated medical costs. However, CM did not affect the number of scheduled appointments attended or drug use. The program designed for this case study was evaluated in relation to the nine special considerations emphasized by Petry [33]. These considerations were examined in order to pinpoint potential explanations for why CM was not effective in changing certain behaviors in this case and to offer suggestions to clinicians in designing CM protocols.

**(i) Behavioral Contracting.**

Although Veteran was provided with a behavioral contract that explained the target behaviors, schedule of monitoring, and contingencies clearly from the perspective of the clinicians, this contract might have been confusing for the Veteran. For example, the contract included two different reinforcement schedules (with different amounts of reinforcement) for two different target behaviors. In addition, the contract states that any aggressive behavior will result in a penalty; this effectively added another target behavior to the contract. Research suggests that targeting one behavior might have larger effects than targeting multiple behaviors [6, 7].

**(ii) Objectively Quantified Behaviors.**

The target behavior of reduced weekend admissions was objectively defined as “not requesting admission to the hospital between 4PM Friday and 8AM Monday.” Outpatient attendance was defined as attending one scheduled outpatient appointment each week with Veteran's case manager. The contract could be even more clear and objective if it specified an appointment time, especially given the fact that Veteran presented for unscheduled appointments two times during the study period.

**(iii) Consistent Monitoring.**

Both target behaviors (outpatient attendance and weekend admissions) were monitored consistently throughout the CM period.

**(iv) Frequency.**

To increase frequency, the contract could have been written to reinforce twice weekly (rather than once weekly) scheduled visits. In addition, if abstinence was a targeted behavior, high-frequency drug testing (e.g., 2-3 times per week) could improve the likelihood of abstinence.

**(v) Successive Approximations.**

In the present case study, successive approximations for target behaviors were not rewarded. For example, we might have successively reinforced attendance by first reinforcing any attendance (e.g., scheduled visits as well as walk-ins) and later only reinforcing attendance at scheduled appointments.

**(vi) Priming.**

This veteran was not “primed” for reinforcement; he had to either avoid a weekend hospital admission or attend an outpatient appointment to receive a voucher. Priming vouchers may be a low-cost method of stimulating interest in the program and could improve outcomes.

**(vii) Immediacy.**

Although the Veteran did receive vouchers immediately upon attendance at outpatient appointments, reinforcement for weekend nonadmittance was often delayed. Coupons were available the Monday following each nonadmitting weekend; however, he often did not pick up earned coupons in a timely manner. This lack of engagement might be related to the issue of magnitude described below.

**(viii) Magnitude.**

In the present case, the magnitude of reinforcers might have been insufficient. Because of his 100% service-connected disability, the Veteran received approximately $2500 per month. Thus, a $1 coupon book might be viewed as insignificant relative to his income. However, research indicates that income level is not related to the effectiveness of CM [34]. Rather, regardless of income, reinforcement magnitude likely needs to be of a sufficient level to spur behavior change. Research (e.g., [35]) suggests a clear relationship between magnitude and effectiveness of CM. In the present case study, the schedule may have been adjusted to a higher magnitude amount by increasing the amount per behavior, increasing the frequency of reinforcement opportunities, increasing the escalation or bonuses, or some combination of these factors. At a minimum, the lower threshold of average earnings per day should be at least $5 per day, as Lussier et al.'s [7] meta-analytic results suggested that studies with lower average daily reinforcement magnitudes were less effective.

Also related to the issue of magnitude, the differential magnitude of reinforcers for the two target behaviors might help explain why CM reduced hospital admissions but did not affect outpatient attendance. For outpatient attendance, Veteran received $1 the first week, increasing by $2 each week. However, for not being admitted on weekends he received $7 the first weekend, and that amount increased by $3 each week.

**(ix) Escalating Reinforcers and Bonuses.**

The current CM protocol did use an escalating system of reinforcement. However, the program could have been improved by modifying it as needed when it was not optimally effective. For example, the schedule could have been modified by increasing the level of reinforcement (i.e., increased voucher values) or using different types of reinforcements (e.g., other resources in the community) that might be more rewarding for the Veteran.

## 4. Discussion

In the present case study, CM was marginally effective for decreasing frequency of weekend hospitalizations but did not affect the percentage of scheduled outpatient appointments attended. However, we cannot conclude that CM does not have the potential to improve treatment attendance. In fact, CM has consistently shown robust positive effects on treatment attendance, even in populations with cooccurring disorders [18, 19, 21, 27, 33]. Rather, as indicated above, the lack of an effect of CM on attendance is more likely reflective of shortcomings in the design and implementation of this CM program. We suggest that making the behavioral contract clearer and more understandable, increasing the reinforcing value of the rewards, incorporating reinforcement for successive approximation of the target behavior (e.g., reinforcing walk-in outpatient attendance), and perhaps using different target behaviors (e.g., abstinence) would likely improve the results of this intervention for this individual.

 CM did not affect results of urine drug screens; the Veteran's drug screens before and during CM were all positive for cocaine use. This result is understandable, given that abstinence was not a targeted behavior in this CM contract. Given the experience with this client, we suggest clinicians target the single behavior most likely to have direct and indirect impacts on the patients' functioning. For example, rather than targeting weekend admission and appointment attendance, we might have first targeted cocaine-negative urine submissions. If sustained abstinence was achieved, the client may have voluntarily reduced admissions or begun attending treatment sessions. An alternate approach may be the reinforcement for completion of activities related to treatment goals [36]. In these CM protocols, the client and clinician collaboratively generate a list of 3-4 activities to be completed before the next visit. Typical activities include attendance of Alcoholics Anonymous meetings, attending a medical appointment, and obtaining or submitting paperwork to obtain housing. As with other CM protocols, the contracted activities must be objectively verifiable in order to earn reinforcement. Given this client's instability in many areas of functioning, the activity-focused CM protocols may have stimulated progress in multiple areas of living.

### 4.1. Recommendations to Clinicians

CM may have utility in patients with substance use and cooccurring disorders, and these effects might be greater when protocols more closely incorporate CM's underlying behavioral principles. Specific incentives for avoiding weekend hospitalization led to marginal reductions in inappropriate attempts for hospital admission in the present study. By reducing unnecessary hospital admissions, CM also served to reduce hospital costs associated with inpatient visits (resulting in cost savings to the medical center of over $5,000 in 3 months). However, CM did not increase the number of scheduled outpatient appointments that the Veteran attended. In addition, CM did not affect results of drug screens, which were not specifically targeted by CM.

Our results highlight challenges in using CM in populations with substance use and cooccurring disorders. In the present study, the Veteran had limited willingness to address psychiatric issues and medication compliance, faced several legal charges, and was homeless. The CM program created for this individual was designed to increase appropriate and consistent use of available hospital resources. However, we recognize several areas for improvement in the behavioral contract used for this individual. Specifically, the behavioral contract might have been overwhelming or confusing for the Veteran, rewards were likely of insufficient magnitude, and rewards were not always provided immediately after target behaviors.

Based on the present study, we emphasize the need for behavioral contracts to: (1) be very clear and understandable, (2) target goals that are most pertinent to the individual at present (in an attempt to target the most critical behaviors without including so many behaviors that the contract is overwhelming or difficult to understand), (3) require frequent and consistent monitoring of behaviors (e.g., drug screens), (4) utilize reinforcers of sufficient magnitude, and (5) provide reinforcers as immediately as possible upon completion of target behaviors. Furthermore, rewards used in CM might have more reinforcement value for patients if hospital systems partner with other services in the community to offer a wider array of rewards in the community, rather than providing coupons that can only be used in a particular hospital setting. Although CM requires a great deal of planning and consistent effort on the part of the treatment team, it may have the potential to improve quality of life among individuals with cooccurring disorders.
